# Risk factors for serious infections in ANCA-associated vasculitis

**DOI:** 10.1136/ard-2022-223401

**Published:** 2023-01-26

**Authors:** Balazs Odler, Regina Riedl, Philipp Gauckler, Jae Il Shin, Johannes Leierer, Peter A Merkel, William St. Clair, Fernando Fervenza, Duvuru Geetha, Paul Monach, David Jayne, Rona M Smith, Alexander Rosenkranz, Ulrich Specks, John H Stone, Andreas Kronbichler

**Affiliations:** 1 Department of Medicine, University of Cambridge, Cambridge, UK; 2 Division of Nephrology, Department of Internal Medicine, Medical University of Graz, Graz, Austria; 3 Institute for Medical Informatics, Statistics and Documentation, Medical University of Graz, Graz, Austria; 4 Department of Internal Medicine IV, Nephrology and Hypertension, Medical University Innsbruck, Innsbruck, Austria; 5 Yonsei University College of Medicine and Severance Children's Hospital, Seoul, Republic of Korea; 6 Division of Rheumatology, University of Pennsylvania, Philadelphia, Pennsylvania, USA; 7 Division of Rheumatology and Immunology, Duke University, Durham, North Carolina, USA; 8 Division of Nephrology and Hypertension, Mayo Clinic, Rochester, Minnesota, USA; 9 Division of Nephrology, Johns Hopkins University, Baltimore, Maryland, USA; 10 VA Boston Healthcare System, West Roxbury, Massachusetts, USA; 11 Division of Pulmonary and Critical Care Medicine, Mayo Clinic, Rochester, Minnesota, USA; 12 Division of Rheumatology Allergy, and Immunology, Massachusetts General Hospital, Harvard Medical School, Boston, Massachusetts, USA

**Keywords:** rituximab, cyclophosphamide, Infections, ANCA, Vasculitis

## Abstract

**Objectives:**

Severe infections contribute to morbidity and mortality in antineutrophil cytoplasm antibody-associated vasculitis (AAV). This study aimed to identify risk factors associated with severe infections in participants of the Rituximab versus Cyclophosphamide for ANCA-Associated Vasculitis (RAVE) trial.

**Methods:**

Data on 197 patients recruited into the RAVE trial were analysed. Participants received either rituximab (RTX) or cyclophosphamide (CYC), followed by azathioprine (AZA). Clinical and laboratory data of patients with and without severe infections (≥grade 3, according to the Common Terminology Criteria for Adverse Events version 3.0) were compared. Risk factors for severe infections were investigated using Cox-regression models.

**Results:**

Eighteen of 22 (82%) severe infections occurred within 6 months after trial entry, most commonly respiratory tract infections (15/22, 68%). At baseline, lower absolute numbers of CD19+ cells were observed in patients with severe infections either receiving RTX or CYC/AZA at baseline, while CD5+B and CD3+T cells did not differ between groups. In Cox-regression analysis, higher baseline serum immunoglobulin M levels were associated with the risk of severe infections, whereby a higher baseline total CD19+B cell number and prophylaxis against *Pneumocystis jirovecii* with trimethoprim-sulfamethoxazole (TMP/SMX) with decreased risk of severe infections. Use of TMP/SMX was associated with lower risk of severe infections in both groups, receiving either RTX or CYC/AZA.

**Conclusions:**

The use of low-dose TMP/SMX is associated with reduced risk of severe infections in patients with AAV treated with either RTX or CYC/AZA. Reduced B cell subpopulations at start of treatment might be a useful correlate of reduced immunocompetence.

**WHAT IS ALREADY KNOWN ON THIS TOPIC**

- Severe infections are the leading cause of death within the first year of diagnosis. Risk factors for severe infections are mainly derived from retrospective analysis, while data from randomised controlled trials are limited.
- Prior analyses focused on cyclophosphamide (CYC)-treated patients, but one retrospective study on patients treated with rituximab (RTX) found a reduced frequency of severe infections when trimethoprim-sulfamethoxazole (TMP/SMX) was used as prophylaxis against *Pneumocystis jirovecii*.

**WHAT THIS STUDY ADDS**

- This study used data from a randomised clinical trial to demonstrate the effectiveness of treatment with low-dose TMP/SMX in patients with antineutrophil cytoplasm antibody-associated vasculitis (AAV), who received RTX or CYC/azathioprine to reduce the incidence of severe infections. TMP/SMX may exhibit additional beneficial effects on respiratory tract infections beyond preventing *Pneumocystis jirovecii* pneumonia.
- At the initiation of treatment, a lower CD19+B cell number is associated with a higher risk of severe infections.

**HOW THIS STUDY MIGHT AFFECT RESEARCH, PRACTICE OR POLICY**

- These findings indicate that the use of extended courses of TMP/SMX may be considered in patients with AAV to prevent severe infections during the induction of remission of vasculitis if favourable risk and benefit is confirmed by future studies.
- The results suggest that baseline B cell subpopulations might be a potential marker of immunocompetence.

## Introduction

The therapeutic landscape in anti-neutrophil cytoplasm antibody (ANCA)-associated vasculitis (AAV) has been expanded during the last decades, including the approval of rituximab (RTX) in the induction of remission of active granulomatosis with polyangiitis (GPA) and microscopic polyangiitis (MPA). The Rituximab versus Cyclophosphamide for ANCA-Associated Vasculitis (RAVE) trial found non-inferiority in the induction of remission of RTX to cyclophosphamide (CYC) as an agent to induce remission and azathioprine (AZA) to maintain remission.^1 2^ During the follow-up period of 6 months, a number of serious infections, defined as National Cancer Institute (NCI) Common Terminology Criteria for Adverse Events (CTCAE) version 3, were balanced between the groups.^1^ Moreover, during the extended follow-up of up to 18 months, the number of trial participants with at least one serious infection also remained balanced between the therapy arms.

Infections are a major contributor to morbidity and mortality of patients with AAV.^3 4^ In the first year of diagnosis, infections are the main cause of death, while infectious complications remain the leading contributing factor to mortality thereafter.^5^ Most risk factors predicting severe infections have been proposed in individuals receiving CYC as induction therapy, including leucopaenia, lymphopaenia, lung disease, severe kidney function impairment and older age,^6^ while more recent data confirmed the association of severe infections and age as well as lung disease in patients receiving RTX.^7^ The use of trimethoprim-sulfamethoxazole (TMP-SMX) in a prophylactic dose to prevent *Pneumocystis jirovecii* pneumonia (PJP), however, reduced the risk of serious infections in patients treated with RTX.^7^

This ancillary study to RAVE analysed risk factors of severe infections reported in the trial for all patients and the two treatment subgroups. The aim of the study was to identify risk factors associated with severe infections in participants of the RAVE trial, including the impact of different prophylactic measures to prevent PJP, and the changes of immune cell subsets (‘immunocompetence’, ie, CD19+ cells at baseline and repopulation after therapy) on the risk to develop serious infectious complications.

## Methods

### Study participants and treatment regimens

Data from the RAVE trial^1 2^ were used for this post hoc analysis. Briefly, the RAVE trial was a multicentre, double-blind, randomised, controlled trial that randomised 197 ANCA positive participants in a 1:1 ratio to receive either RTX (N=99, 375 mg/m^2^ each week for 4 weeks) or daily oral CYC (N=98, 2 mg/kg, adjusted for kidney function for 3–6 months) followed by a maintenance therapy with AZA (2 mg/kg, up to 150 mg/day). Patients in both groups received the same glucocorticoid (GC) protocol consisting of 1–3 days intravenous methylprednisolone pulses, followed by prednisone taper with scheduled discontinuation by 6 months in case remission was achieved without disease flares. Methylprednisolone could be administered during the screening/randomisation period and the baseline evaluations had to be completed within 14 days of the first dose of RTX/placebo, that means that some patients received methylprednisolone pulses before study medication was started. Participants in both groups received daily prophylaxis against PJP with single-strength or double-strength TMP/SMX, or dapsone (100 mg/day) or atovaquone (750 mg two times per day) if allergies were present. As stated in the study protocol, PJP prophylaxis was continued while participants were receiving study medications, GC or other immunosuppressive drugs under best medical judgement. Participants treated with open-label RTX had to undergo PJP prophylaxis for 6 months after their last RTX infusion.^1 2^ All patients provided consent for the use of both clinical and laboratory data collected during the trial and for subsequent ancillary studies.

### Definition of severe infections

Infections were collected during the entire clinical trial and defined by the NCI CTCAE version 3.0. Infections graded 3 or higher were considered severe infections, and grades 1 or 2 as mild infections. The analysis included severe infections due to possibly lacking data on mild infections not necessitating additional medical treatment and a general under-reporting in clinical practice. Infection sites were grouped as respiratory tract (RTI), gastrointestinal (GI), urinary tract (UTI) and other infections.

### Clinical data

The following data were obtained: age, gender, weight, height, body mass index, Birmingham Vasculitis Activity Score/WG at baseline, ANCA serotype (anti-myeloperoxidase (MPO) or anti-proteinase 3 (PR3)), disease phenotype (GPA, MPA), disease flare extent (limited or severe), organ involvement (kidney, skin, eye, subglottic, lung, ear, nose and throat, heart, peripheral nervous, sensorineural deafness), fever (>38°C), arthralgia or pulmonary haemorrhage, data on new diagnosis or relapsing disease, immunosuppressive therapy (methylprednisolone bolus, cumulative doses of GC, CYC and RTX) and use of antibiotics as prophylaxis against PJP (TMP-SMX, dapsone, atovaquone and no prophylaxis). Laboratory data included serum creatinine, white cell count, leucocyte count, total Ig and Ig isotypes (IgA, IgG, IgM) and data on B and T cell subsets (CD3, CD5, CD19) at different study time points.

### Statistical analysis

Continuous parameters are summarised as the median and range (minimum, maximum) and categorical parameters are presented as absolute and relative frequencies. Characteristics at baseline and CD3, CD5 and CD19 at different study time points were compared between patients with severe infections and no severe infection by Mann-Whitney U and Fisher’s exact test. To evaluate risk factors for first severe infection within 18 months after therapy started, univariable Cox proportional hazard regression analyses were performed. In a multivariable Cox regression model, all parameters with a p<0.1 in univariable analysis were included and backward selection was used in the final model. Results are presented as HRs with corresponding 95% CIs. All analyses were performed for the whole cohort and stratified for the treatment groups (RTX and CYC/AZA), except for the multivariable analysis due to the small number of events. Additionally, Kaplan-Meier curves for infection-free survival are presented for patients with and without use of TMP/SMX. Missing data were not imputed. A p<0.05 was considered statistically significant. The results are interpreted exploratory and no correction for multiple testing was performed. All analyses were performed by using SAS V.9.4 (SAS Institute).

## Results

### Baseline patient characteristics stratified by infectious complications

There were twenty-two patients with at least one severe infection within the first 18 months of the trial. Median follow-up time was 531 days (2–581 days). Baseline characteristics between patients with and without severe infections were similar; however, more patients without severe infection received prophylaxis against PJP with TMP/SMX (94% vs 73%, p=0.005). At least one dose of methylprednisolone was administered in 180 (91.4%) of randomised patients. Two severe infections (6.3%) occurred in 32 patients receiving a total of ≥3000 mg of methylprednisolone. In addition, patients with a severe infection had lower median CD19+B cell number at baseline (total cell number; 120.5/µL (range 12.3–759.1) vs 219.1/µL (range 2.4–1282.1), p=0.001) (table 1). Relapsing patients had a lower CD19+B cell number at baseline (173.5/µL (range 2.4–1282.1) vs 219.1/µL (range 24.9–1279.8), p=0.012), while there was no difference whether patients were exposed to CYC before enrolment into the RAVE trial or not (154.3/µL (range 2.4–1282.1) vs 203.8/µL (range 32.1–655), p=0.274) (online supplemental table 1). Fifteen (68.2%) out of 22 severe infections occurred in relapsing patients at baseline. Among those patients, 12 (80%) received therapy with immunosuppression/cytotoxic agent before trial inclusion. Further patient characteristics are summarised in table 1, while differences in patients receiving either RTX or CYC/AZA and patients with and without TMP/SMX use are shown in online supplemental tables 2 and 3.

10.1136/ard-2022-223401.supp1 Supplementary data

**Table 1:** Baseline characteristics of patients without and with severe infections

	Patients without severe infections (N=175)	Patients with severe infections (N=22)	P value
Age (years)	53 (15–92)	52 (30–80)	0.863
Female sex	87 (50)	10 (46)	0.822
BMI (kg/m^2^)	27.6 (15.7–51.7)	28.3 (21.8–41.3)	0.290
Baseline BVAS/WG (overall score)	8 (3–23)	8 (3–16)	0.397
Diagnosis at enrollment			
Newly diagnosed at enrollment	89 (51)	7 (32)	0.115
Relapsing disease	86 (49)	15 (68)	
Disease phenotype			
GPA	131 (75)	16 (73)	0.300
MPA	43 (25)	5 (23)	
Indeterminate	1 (1)	1 (5)	
ANCA serotype			
PR3	118 (67)	13 (59)	0.476
MPO	57 (33)	9 (41)	
Pulmonary haemorrhage	47 (27)	4 (18)	0.450
Organ involvement			
Kidney	116 (66)	15 (68)	1
Lung	80 (46)	80 (46)	0.498
Heart	1 (1)	0 (0)	1
Ear, nose and throat	97 (55)	11 (50)	0.656
Mucous membranes and eyes	40 (23)	6 (27)	0.603
Cutaneous	31 (18)	3 (14)	0.772
Severe disease flare	39 (22)	2 (9)	0.262
Use of TMP/SMX	164 (94)	16 (73)	**0.005**
Use of dapsone	6 (3)	5 (23)	
Use of atovaquone	1 (1)	1 (5)	
None	4 (2)	0 (0)	
Laboratory results			
Creatinine (mg/dL)	1.1 (0.5–4.2)	1.2 (0.7–3.1)	0.594
Lymphocytes (/µL)	1300 (300–12200)	1400 (500–3900)	0.933
WCC (/µL)	11.2 (2.3–25.8)	11.9 (4.6–26.9)	0.887
Total Ig (mg/dL)	1174 (409–3101)	1203 (580–2008)	0.619
IgA (mg/dL)	153 (14–882)	188 (49–289)	0.357
IgG (mg/dL)	907 (322–2598)	907 (493–1585)	0.935
IgM (mg/dL)	84 (16–271)	86 (29–716)	0.595
Total CD3+T cells	729 (49–3656)	751 (51–2577)	0.246
Total CD19+B cells	219 (2–1282)	121 (12–759)	0.001
Total CD5+B cells	17 (0–422)	10 (1–139)	0.259

Continuous variables are expressed as median (minimum and maximum). Categorical variables are n (%).

ANCA, antineutrophil cytoplasmic antibody; BMI, body mass index; BVAS, Birmingham Vasculitis Activity Score; GPA, granulomatosis with polyangiitis; MPA, microscopic polyangiitis; MPO, myeloperoxidase; PR3, proteinase 3; TMP/SMX, trimethoprim-sulfamethoxazole; WCC, white cell count.

### Characteristics of severe infections

**Figure 1:**
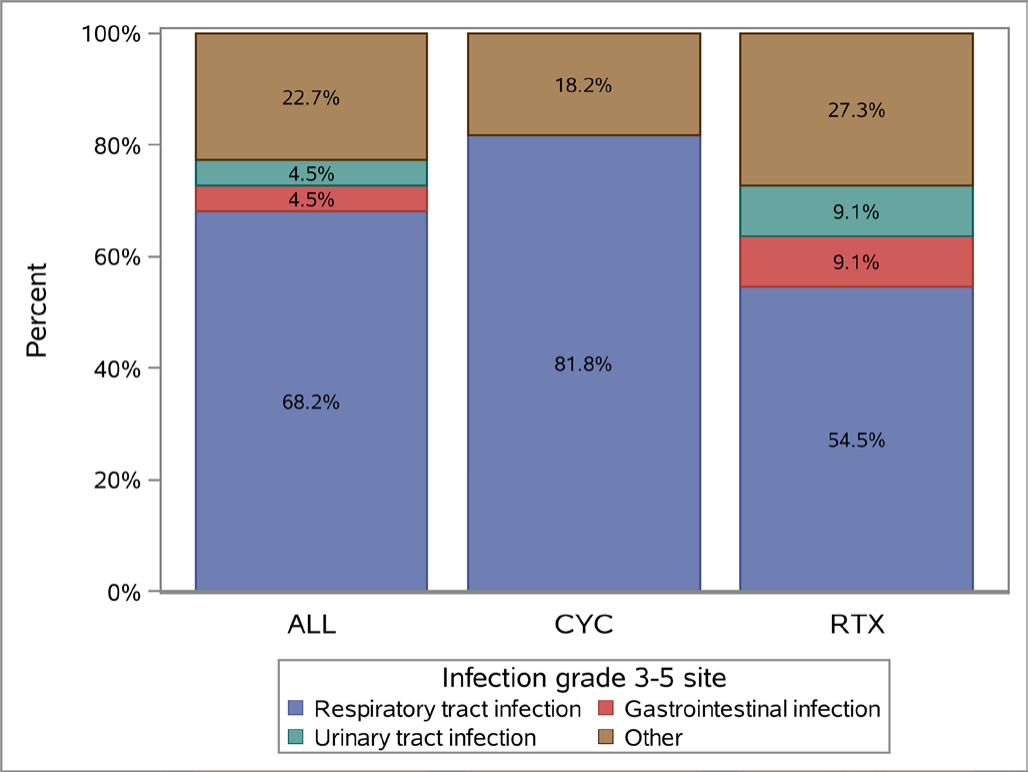
The distribution of severe infection sites in the whole study cohort. CYC, cyclophosphamide; RTX, rituximab.

The most common infections were RTI (15/22, 68.2%), followed by GI (1/22, 4.6%), UTI (1/22, 4.6%) and other infections (5/22, 22.7%, figure 1). Other infections included joint and skin infections as well as abdominal abscess, neutropenic fever and poststreptococcal glomerulonephritis. Most of the severe infections were grade 3 (18/22, 81.8%), with a grade 4 infection recorded in 3 (13.6%) patients, and a grade 5 infection in 1 patient (1/22, 2.1%). Eighteen of 22 (81.8%) severe infections occurred within 6 months after trial entry. During the whole study period the median time to the first severe infection was 85 days (range: 2–513). Patients in the CYC/AZA group experienced a severe infectious complication earlier as compared with those in the RTX group (39 (13–414) vs 126 (2–513) days, respectively, p=0.076).

### Predictors of severe infections

**Figure 2:**
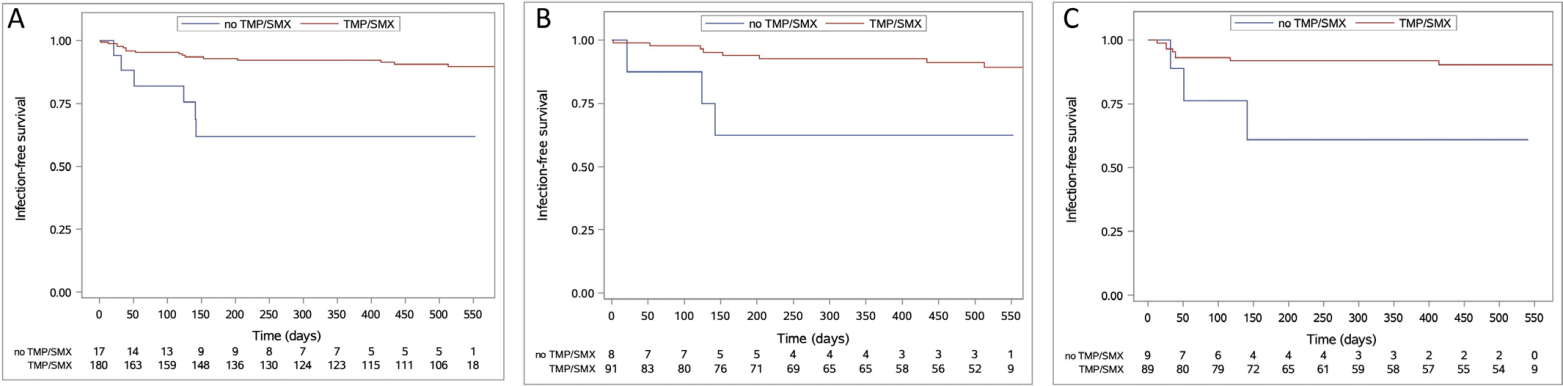
Kaplan-Meier curves for infection-free survival in patients receiving trimethoprim-sulfamethoxazole (TMP/SMX) prophylaxis in the whole study cohort (A) and stratified by treatment regimen (B) rituximab; (C) cyclophosphamide/azathioprine.

Univariable Cox regression analysis was performed to identify clinical and laboratory predictors of severe infectious complications during the study period (online supplemental table 4). Total CD19+B cell number, serum IgM and TMP/SMX prophylaxis were significantly associated with severe infections and were thus included into the multivariable analysis. In the multivariable analysis, higher baseline serum IgM levels were associated with increased risk of severe infections (HR: 1.005; 95% CI: 1.002 to 1.009; p=0.006), whereby a higher baseline total CD19^+^ B cell number (HR: 0.995, 95% CI: 0.991 to 0.999; p=0.011) and TMP/SMX pophylaxis (HR: 0.232; 95% CI: 0.087 to 0.623; p=0.004; figure 2A) with decreased risk of severe infections. Results of the multivariable analysis are shown in table 2.

**Table 2:** Multivariable COX regression analysis of predictors of severe infections

Covariate	HR	95% CI	P value
Total CD19^+^ B cells	0.995	0.991 to 0.999	0.011
Serum IgM	1.005	1.002 to 1.009	0.006
Use of TMP/SMX	0.232	0.087 to 0.623	0.004

TMP/SMX, trimethoprim-sulfamethoxazole.

### Use of TMP/SMX and risk of severe infections

Seventeen patients (8.6%) did not receive TMP/SMX as prophylaxis against PJP (ie, for lack of compliance or having other prophylactic measures). Among these, six patients (35.3% in total; 3 in the RTX and CYC/AZA arms each, respectively) experienced a severe infection. The use of TMP/SMX remained associated with a lower risk of severe infections in both treatment subgroups (RTX or CYC/AZA): (HR: 0.204; 95% CI: 0.054 to 0.772; p=0.019 and HR: 0.232; 95% CI: 0.061 to 0.884; p=0.032; respectively; figure 2B, C).

### T and B cell subsets and risk of severe infections in follow-up

**Figure 3:**
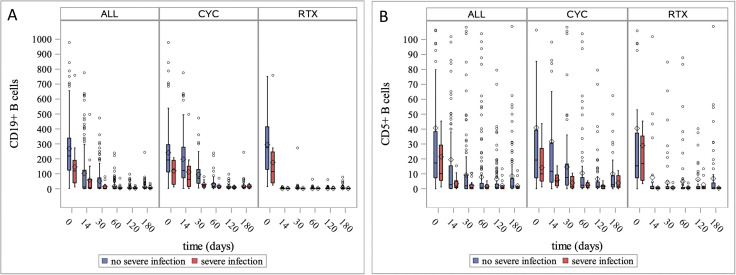
Box plots for peripheral CD19+ (A) and CD5+ (B) B cells during the first 180 days after therapy initiation in the whole study cohort and stratified by treatment regime. Diamond indicates the mean value.

Data on peripheral CD3+T cells, as well as CD19+ and CD5+ B cells were analysed at baseline and during the first 6 months of follow-up. Patients with severe infections had a lower total number of CD19+B cells at baseline (as described previously; table 1) and tended to have lower CD5+B cells at month 1 (0.7/µL (range: 0.0–10.5) vs 1.9/µL (range: 0.0–108.3); p=0.053; figure 3A, B), while CD3+T cells did not differ between groups. A lower total number of CD19+B cells at baseline and at month one was observed in patients with severe infections compared to patients without severe infections in the RTX arm (114.8/µL (range: 25.7–759.1) vs 276.7/µL (range: 14.0–1282.1); p=0.022 and 0.6/µL (range: 0.2–3.5) vs 1.9/µL (range: 0.0–273.0); p=0.005; respectively; figure 3A), while no differences in the CD5+B cells and CD3+T cells were observed. In the CYC/AZA arm, patients with severe infections had lower total number of CD19+B cells at baseline and month 1 (128.7/µL (range: 12.3–207.7) vs 193.6 (range: 2.4–977.4); p=0.023 and 25.0/µL (range: 7.5–81.7) vs 72.0 (range: 4.2–472.7); p=0.011; respectively, figure 3A), as well as CD5+B cells at week two after therapy initiation (4.8/µL (range: 0.8–15.5) vs 11.7/µL (range: 0–508.7); p=0.032 figure 3B). Additionally, a tendency to lower total number of CD3+T cells at baseline and week two were observed in the CYC/AZA study group (p=0.095 and p=0.069; respectively).

## Discussion

Using data from the RAVE trial, this study identified independent clinical and immunologic predictors associated with severe infections in patients with AAV. Patients treated with either GCs and RTX or CYC/AZA without use of TMP/SMX for prophylaxis against PJP were more likely to experience severe infections, especially within the first 6 months after initiation of therapy. Furthermore, a lower absolute number of CD19+ in both therapeutic arms at baseline predicted serious infections.

Since infections are one of the leading causes of death in patients with AAV,^3 4 8 9^ strategies to prevent or minimise infections are of importance. Several international guidelines on management of AAV recommend prophylaxis against PJP infection in patients receiving either RTX or CYC.^10–15^ Nevertheless, most of these recommendations are based on data from observational studies. This post hoc analysis of the RAVE trial is the first analysis of data derived from a randomised controlled trial providing evidence for the role of low-dose TMP/SMX in preventing severe infections in patients with AAV. These results reveal a significant benefit in patients receiving either RTX or CYC/AZA. A retrospective analysis found that the prophylactic use of TMP/SMX was associated with a lower rate of severe infection in patients receiving RTX.^7^ In addition, a recent study by Waki *et al* reported similar protective effects of TMP/SMX on serious infections within the first 6 months of follow-up, however, differences in therapy modalities discriminating CYC and RTX were not analysed.^16^ In both studies, RTI were the most frequent infectio (68.4% and 33.3%; respectively), including a low incidence of PJP (1/69 and 2/15 of the reported RTI; respectively), which is consistent with the current analysis (15/22, 68% RTI; 1/15 (7%) with a PJP infection). These results suggest that the use of TMP/SMX may exhibit additional beneficial effects on RTI beyond preventing PJP. In the RAVE trial, single-strength or double-strength TMP/SMX was given as long as patients received study medications, including GC or other immunosuppressive therapy under best medical judgement.^1 2^ Since most of the infections occurred in the first 6 months after therapy initiation, withdrawal after cessation of immunosuppression may be useful as the risk of severe adverse events possibly associated with the use of TMP/SMX is substantial.^17 18^ Nevertheless, a randomised controlled trial addressing this question is necessary, that is, looking at differences of prophylaxis prescribed for 6 months in comparison to a prolonged prescription during long-term follow-up.

Analysis of risk factors predicting infectious complications has been performed, mainly in observational studies, where various clinical and serological prognostic factors have been reported.^6 19–23^ In the RAVE cohort, baseline serum IgM levels and total number of CD19+B cells were independently associated with severe infections in the multivariable analysis. Serum IgM is the first antibody secreted after exposure to foreign antigens and is effective at engaging complement to pathogens.^24^ Patients with selective IgM deficiency are prone to develop serious infections and autoimmune conditions.^24^ Among patients with AAV, serum IgM hypogammaglobulinaemia after therapy with RTX is well described. Nonetheless, conflicting evidence exists whether or not low serum IgM levels are associated with severe infections in AAV. In our cohort, a higher serum level of IgM was associated with the risk of severe infections. The interpretation of this finding is speculative and further studies are necessary to characterise this potential association in detail, including serial measurements of IgM serum levels, and especially measurement of IgM at the time of infection rather than at baseline. The observed association between severe infections and baseline total CD19+B cell number was not unexpected. Importantly, it might draw further attention to careful monitoring of patients with AAV who initially have low CD19+B cells irrespective of the modality of induction regime used. In addition, soluble immune checkpoints (sCD27, sTim-3 and sBTLA) predicted infectious complications and relapses,^25^ and the combination of baseline CD19+B cells with those biomarkers may be more accurate to predict severe infections and future relapses, especially in patients receiving RTX as a remission induction therapy. Together, these markers might be used to monitor immunocompetence of individuals at baseline and may help to tailor immunosuppression.

The administration of intravenous methylprednisolone was protocolised in the RAVE trial and reasons that no methylprednisolone was administered in 17 patients remain obscure. Only two patients with a total dose of ≥3000 mg of methylprednisolone had a severe infection. Further analyses of methylprednisolone dosing might be biased as patients at risk to develop severe infections (ie, with diabetes mellitus, or older age) might only have received one pulse and this strategy was most frequently used in the RAVE trial. In general, a reduced-dose GC protocol as used in the PEXIVAS,^26^ LoVAS^27^ and RITAZAREM^28^ trials seems to be favourable in terms of reduction of severe infections.

The effect of CYC on B cell subpopulations has been previously described. Zhu *et al* found a selective suppression of B cell function in a small cohort of vasculitis patients^29^ who were treated with low dose CYC. In patients with AAV receiving CYC, a depletion of especially naïve B cells was described.^30^ The current study results revealed lower total CD19+ and CD5+ B cell numbers during the first month after therapy initiation in patients treated by CYC. These results suggest that CYC therapy does not lead to a permanent loss of humoral immunity, nevertheless the assessment of B cell subpopulations before therapy initiation and early thereafter might be useful to identify patients with AAV at higher risk for severe infections irrespective of treatment regimen.

This study has certain limitations. The relatively low number of severe infections observed during the study period may lead to underestimation of certain prognostic factors for infectious complications. Moreover, the duration and potential adverse events related to the use of TMP/SMX were not recorded in detail and most side effects such as liver function abnormalities or cytopaenias might also occur with other prescribed therapies. Despite these limitations, this study provides important data on severe infectious complications among AAV patients followed in a randomised controlled trial.

In conclusion, this study demonstrates that the use of TMP/SMX as prophylaxis against PJP is significantly associated with reduced risk of severe infections in patients with AAV, independent of the choice of the therapy used to induce remission. These data further suggest that certain B cell subpopulations may serve as a useful tool to determine general immunocompetence of AAV patients, and to identify individuals at higher risk of infectious complications. T results warrant independent confirmation in future studies, with a special focus on ideal duration of TMP/SMX prophylaxis, and exploration of a similar impact of TMP/SMX in the management of patients with other autoimmune diseases.

## Data Availability

Data are available in a public, open access repository. Data are available on itntrialshare.org.
