# Supplementary material for: Risk factors for serious infections in ANCA-associated vasculitis
Source: Ann Rheum Dis. 2023 Jan 26;82(5):681–7. doi: 10.1136/ard-2022-223401 (PMC10176387; doi:10.1136/ard-2022-223401)
Supplement: Supplementary data [file ard-2022-223401supp001.pdf]

Supplementary Table 1. CD19+ B cell count according to baseline characteristics (relapsing versus new onset disease) and the choice of pre-treatment.

	Number of patients	Median (minimum-maximum)	p-value
**Relapsing disease**	101	173.5 (2.4-1282.1)	0.012
**New diagnosis**	96	219.1 (24.9-1279.8)	
**No exposure to cyclophosphamide**	22	203.8 (32.1-655)	0.274
**Prior exposure to cyclophosphamide**	79	154.3 (2.4-1282.1)	

Supplementary Table 2. Baseline characteristics of patients with and without severe infections stratified by treatment regime

	**CYC**			**RTX**		
	**Patients without severe infections (N=87)**	**Patients with severe infections (N=11)**	**p-value**	**Patients without severe infections (N=88)**	**Patients with severe infections (N=11)**	**p-value**
**Age (years)**	52 (15-79)	52 (40-80)	0.277	56 (16-92)	50 (30-73)	0.518
**Female**	41 (47)	4 (36)	0.541	46 (52)	6 (55)	1
**BMI (kg/m^2)**	28.7 (18.9-51.7)	27.0 (23.6-33.2)	0.485	26.5 (15.7-47.2)	31.4 (21.8-41.3)	0.032
**Baseline BVAS (overall score)**	8 (3-23)	7 (4-16)	0.683	8 (3-19)	8 (3-11)	0.440
**Newly diagnosed at enrollment**	44 (51)	4 (36)	0.530	45 (51)	3 (27)	0.202
**Disease phenotype**						
GPA	66 (76)	8 (73)	1	65 (74)	8 (73)	0.316
MPA	21 (24)	3 (27)		22 (25)	2 (18)	
Indeterminate	0 (0)	0 (0)	-	1 (1)	1 (9)	
**ANCA serotype**						
PR3	59 (68)	6 (55)	0.500	59 (67)	7 (64)	1
MPO	28 (32)	5 (46)		29 (33)	4 (36)	
**Pulmonary hemorrhage**	22 (25)	1 (9)	0.450	25 (28)	3 (27)	1
**Organ involvement**						
Renal	56 (64)	10 (91)	0.100	60 (68)	5 (46)	0.180
Lung	44 (51)	3 (27)	0.204	36 (41)	5 (46)	0.758
Heart	1 (1)	0 (0)	1	0 (0)	0 (0)	-
Ear, Nose and Throat	46 (53)	4 (36)	0.352	51 (58)	7 (64)	1
Mucous membranes and eyes	20 (23)	3 (27)	0.716	20 (23)	3 (27)	0.714
Cutaneous	14 (16)	1 (9)	1	17 (19)	2 (18)	1
**Severe disease flare**	20 (23)	1 (9)	0.448	19 (22)	1 (9)	0.454
**Cumulative CYC dosage (g)**	15.6 (0.6-33.1)	12.7 (1.8-24.5)	0.422	-	-	-
**Cumulative RTX dosage (g)**	-	-	-	2.8 (2.2-3.7)	3.1 (2.5-3.6)	0.216

**TMP/SMX prophylaxis**	81 (93)	8 (73)	0.061	83 (94)	8 (73)	0.043
**Laboratory results**						
Creatinine (mg/dl)	1.1 (0.5-3.4)	1.3 (0.9-3.1)	0.243	1.2 (0.6-4.2)	1.2 (0.7-2.7)	0.700
Lymphocytes (/µl)	1300 (300-12200)	1200 (500-3900)	0.625	1300 (600-4600)	1600 (500-3300)	0.642
WBC (/µl)	11 (3-24)	13 (9-27)	0.071	12 (2-26)	9 (5-24)	0.137
Total Ig (mg/dl)	1214 (409-3101)	1109 (580-1885)	0.479	1134 (540-2776)	1264 (889-2008)	0.126
IgA (mg/dl)	153 (14-882)	186 (49-278)	0.760	154 (54-634)	232 (107-289)	0.413
IgG (mg/dl)	921 (322-2598)	874 (493-1248)	0.394	879 (373-2123)	920 (745-1585)	0.370
IgM (mg/dl)	88 (16-231)	72 (38-359)	0.457	82 (18-271)	117 (29-716)	0.180
Total CD3+ T cells	736 (122-2304)	676 (51-1103)	0.095	676 (49-3656)	829 (133-2577)	0.953
Total CD19+ B cells	194 (2-977)	129 (12-208)	0.023	277 (14-1282)	115 (26-759)	0.022
Total CD5+ B cells	19 (0-261)	10 (1-44)	0.170	15 (0-422)	17 (4-139)	0.796

Continuous variables are expressed as median (minimum and maximum). Categorical variables are n (%).
Abbreviations: ANCA: antineutrophil cytoplasmic antibody; BMI: body mass index; BVAS: Birmingham Vasculitis Activity Score; CYC: cyclophoshamide; GPA: granulomatosis with polyangiitis; Ig: immunoglobulin; MPA: microscopic polyangiitis; MPO: myeloperoxidase; PR3: proteinase 3; RTX: rituximab; TMP/SMX: trimethoprim-sulfamethoxazole; WBC: white blood cells

Supplementary Table 3. Baseline characteristics of patients without and with trimethoprim-sulfamethoxazole use.

	**Patients without TMP/SMX (N=17)**	**Patients with TMP/SMX (N=180)**	**p-value**
**Age (years)**	52 (34-73)	53.5 (15-92)	0.428
**Female**	10 (59)	87 (48)	0.455
**BMI (kg/m^2)**	28.3 (21.8-40.4)	27.6 (15.7-51.7)	0.563
**Baseline BVAS/WG (overall score)**	10 (3-16)	8 (3-23)	0.158
**Newly diagnosed at enrollment**	5 (29)	91 (51)	0.128
**Disease phenotype**			
GPA	14 (82)	133 (74)	0.096
MPA	2 (12)	46 (26)	
Indeterminate	1 (6)	1 (1)	
**ANCA serotype**			
PR3	7 (41)	124 (69)	0.030
MPO	10 (59)	56 (31)	
**Pulmonary hemorrhage**	5 (29)	46 (26)	0.774
**Organ involvement**			
Renal	11 (65)	120 (67)	1

Lung	7 (41)	81 (45)	0.804
Heart	0 (0)	1 (1)	1
Ear, Nose and Throat	10 (59)	98 (54)	0.803
Mucous membranes and eyes	2 (12)	44 (24)	0.369
Cutaneous	6 (35)	28 (16)	0.084
**Severe disease flare**	4 (24)	37 (21)	0.758
**Laboratory results**			
Creatinine (mg/dl)	1.0 (0.6-4.2)	1.2 (0.5-4.1)	0.476
Lymphocytes (/µl)	1200 (600-3300)	1300 (300-12200)	0.906
WBC (/µl)	13 (5-27)	11 (2-26)	0.338
Total Ig (mg/dl)	1230 (544-1885)	1174 (409-3101)	0.855
IgA (mg/dl)	179 (71-278)	155 (14-882)	0.709
IgG (mg/dl)	913 (393-1248)	904 (322-2598)	0.903
IgM (mg/dl)	97 (32-716)	83 (16-271)	0.414
Total CD3+ T cells	688 (133-2577)	730 (49-3656)	0.933
Total CD19+ B cells	191 (26-707)	200 (2-1282)	0.341
Total CD5+ B cells	6 (0-75)	18 (0-422)	0.048

Continuous variables are expressed as median (minimum and maximum). Categorical variables are n (%).
Abbreviations: ANCA: antineutrophil cytoplasmic antibody; BMI: body mass index; BVAS: Birmingham Vasculitis Activity Score; GPA: granulomatosis with polyangiitis; Ig: immunoglobulin; MPA: microscopic polyangiitis; MPO: myeloperoxidase; PR3: proteinase 3; TMP/SMX: trimethoprim-sulfamethoxazole; WBC: white blood cell count.

Supplementary Table 4. Unadjusted univariate Cox regression analysis on the predictors of severe infections

Covariate	Hazard ratio	95% confidence interval	p-value
Age (years)*	1.005	0.977-1.033	0.736
Age > 60 years*	1.031	0.420-2.530	0.946
Gender	1.218	0.526-2.820	0.645
Weight (kg)*	1.004	0.985-1.024	0.674
Height (cm)*	1.001	0.961-1.042	0.962
BMI (kg/m^2)*	1.016	0.955-1.081	0.624
BMI (kg/m^2) > 35*	1.054	0.357-3.115	0.924
Newly diagnosed at enrollment	0.446	0.182-1.095	0.078
Cumulative CYC dosage (mg)	1.000	1.000-1.000	0.249
RTX	0.979	0.424-2.259	0.961
Cumulative RTX dosage (mg)	1.001	1.000-1.003	0.156

RTX dosage at time of relapse (mg)	1.002	0.998-1.007	0.352
RTX at relapse	0.347	0.081-1.493	0.155
Limited disease flare*	0.973	0.381-2.488	0.955
Severe disease flare*	0.429	0.099-1.846	0.255
First disease flare prior to 6 months	0.438	0.102-1.875	0.266
GPA*	1.090	0.399-2.976	0.866
Indeterminate#*	7.398	0.862-63.470	0.068
PR3 serotype*	0.762	0.326-1.784	0.532
BVAS*	0.950	0.823-1.096	0.480
BVAS > 8*	0.753	0.316-1.795	0.522
Fever > 38°C*	0.751	0.254-2.219	0.604
TMP/SMX prophylaxis	0.208	0.081-0.534	**0.001**
Pulmonary hemorrhage*	0.628	0.212-1.854	0.399
Renal disease*	1.071	0.436-2.627	0.881
Arthralgia*	0.631	0.273-1.461	0.283
Lung involvement*	0.666	0.279-1.589	0.360
Peripheral neuropathy*	1.240	0.457-3.361	0.672
ENT involvement*	0.789	0.342-1.820	0.579
Mucous membranes and eyes*	1.292	0.506-3.302	0.593
Cutaneous*	0.699	0.207-2.362	0.564
Subglottic involvement*	0.824	0.111-6.127	0.850
Creatinine (mg/dl)*	1.026	0.594-1.772	0.926
Creatinine > 1.5 mg/dl*	0.840	0.329-2.147	0.716
Combination of age and baseline creatinine*			
Age>60, creatinine <=1.5	0.645	0.184-2.265	0.494
Age<=60, creatinine >1.5	0.437	0.099-1.938	0.276
Age>60, creatinine >1.5	1.212	0.395-3.719	0.737
Lymphocytes (/µl)*	1.000	1.000-1.000	0.979
Lymphocytes < 800 (/µl)*	0.850	0.199-3.638	0.827
WBC (/µl)*	1.016	0.934-1.105	0.710
Total CD3 T cells*	0.999	0.999-1.000	0.207
Total CD19 B cells*	0.995	0.992-0.999	**0.010**
Total CD5 B cells*,*1	0.992	0.979-1.005	0.210
Serum total Ig (mg/dl)*	1.000	0.999-1.001	0.921
Serum IgA (mg/dl)*	1.000	0.996-1.003	0.975
Serum IgG (mg/dl)*	1.000	0.999-1.001	0.743
Serum IgG > 7mg/dL*	0.849	0.287-2.510	0.768
Serum IgG > 5mg/dL*	0.700	0.094-5.213	0.728
Serum IgM (mg/dl)*	1.006	1.002-1.010	**0.002**

Statistically significant p-values appear in boldface type ($p < 0.05$). Clinical and laboratory data correspond baseline values.

Abbreviations: ANCA: antineutrophil cytoplasmic antibody; BMI: body mass index; BVAS: Birmingham Vasculitis Activity Score; CYC: cyclophoshamide; ENT: Ear, Nose and Throat; GPA: granulomatosis with polyangiitis; Ig: immunoglobulin; PR3: proteinase 3; RTX: rituximab; TMP/SMX: trimethoprim-sulfamethoxazole; WBC: white blood cells

[*] At baseline; [*1] 7 days after therapy initiation; [#] n=2
